# Supplementary material for: Genome-Wide Identification and Transcriptional Expression Profiles of Transcription Factor WRKY in Common Walnut (Juglans regia L.)
Source: Genes (Basel). 2021 Sep 19;12(9):1444. doi: 10.3390/genes12091444 (PMC8466090; doi:10.3390/genes12091444)
Supplement: Supplementary file 1 [file genes-12-01444-s001.zip › Supplementary Figures.pdf]

Supplementary materials

Figures:

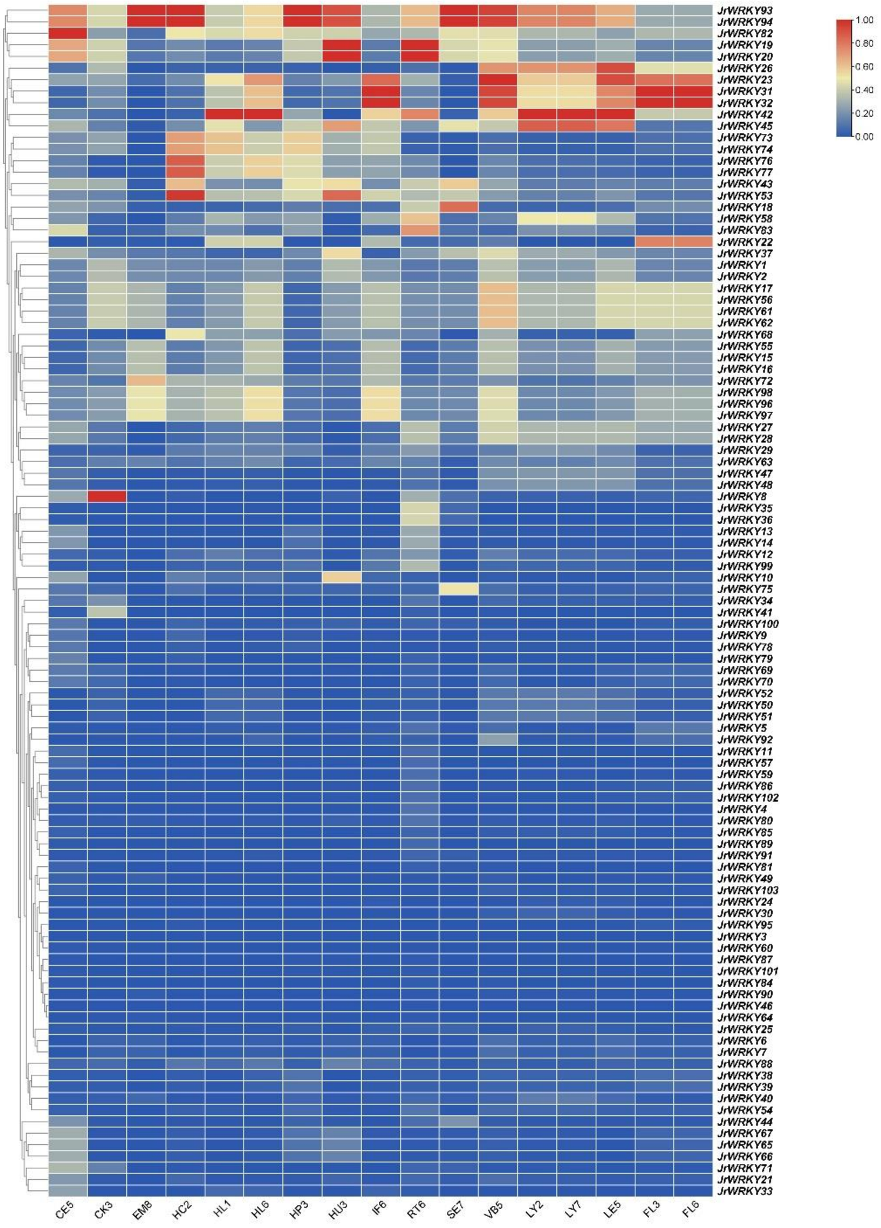

Figure S1. Expression profiles of the walnut *WRKY* genes among 17 tissues. The transcriptome information was seen in (Table S1). The expression level shows from blue to red represent low to high as shown in the right ruler.

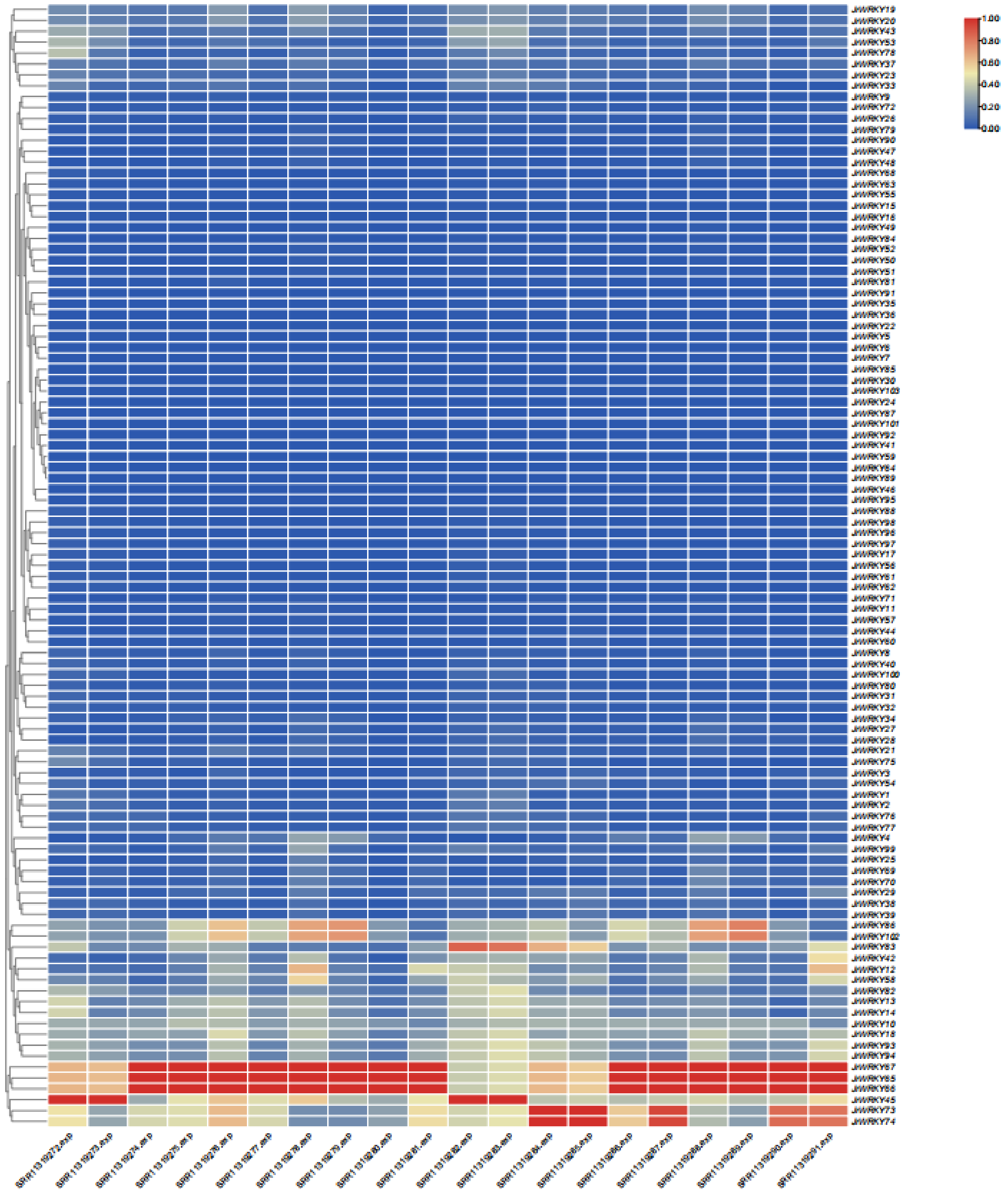

Figure S2. Expression profiles of the walnut *WRKY* genes between anthracnose-resistant F26 and anthracnose-susceptible F423 were used in this study. A total of 10 anthracnose-resistant walnuts and 10 anthracnose-susceptible F423 walnuts (Table S1). The expression level shows from blue to red represent low to high as shown in the right ruler.
